# Chemical priming of strawberry plants under deficit irrigation enhances yield efficiency and physiological resilience

**DOI:** 10.1038/s41598-025-29763-z

**Published:** 2025-12-01

**Authors:** Egli C. Georgiadou, Anna Maria Taliadorou, Eleni D. Myrtsi, Sofia Torrado, Nicolas Valanides, Lorenzo Bini, George A. Manganaris, Vasileios Fotopoulos

**Affiliations:** 1 Department of Agricultural Sciences, Biotechnology & Food Science, Cyprus University of Technology, Lemesos, 3603 Cyprus; 2 Department of Agriculture, Food, Environment, and Forestry (DAGRI), University of Florence, Viale delle Idee 30FI, Sesto Fiorentino, Florence, 50019 Italy

**Keywords:** *Fragaria* x ananassa, Abiotic stress, Deficit irrigation, Drought stress, Stress alleviation, Priming, Proline, Melatonin, Sodium alginate, Physiology, Plant sciences

## Abstract

Strawberry is a high-value crop with substantial water requirements, rendering it particularly vulnerable to irrigation limitations, especially in regions like the Mediterranean, which is characterized by low summer rainfall and restricted water sources. This study investigated the effects of different priming agents (PAs) on yield efficiency and physiological and biochemical performance of strawberry (*Fragaria* x *ananassa* Duch.) cv. ‘Red Sayma 1075’ grown under deficit irrigation. The experiment was conducted under a commercial strawberry setup in Cyprus, using a randomized complete block design (RCBD) with five blocks serving as biological replicates. The treatments were hydroprimed, 100 µM melatonin (Mel), 0.1% w/v sodium alginate (NaA), 0.1% w/v NaA/100 µM Mel, 2 mM proline, and 0.1% w/v NaA/2 mM proline. Three treatment applications were performed at different times, one at root level of tray plants prior to transplantation and two foliar applications at 8 d and 15 d after transplantation. Plants received 50% of standard irrigation following priming applications to simulate conditions of deficit irrigation. Results demonstrated that Mel, NaA, and NaA/Proline treatments significantly enhanced cumulative yield of early harvest compared with hydroprimed controls. Mel and NaA/Mel displayed significantly higher endogenous Mel content compared with controls. In addition, an array of analysis was conducted ar leaf level at 2, 15 and 29 d after imposition of deficit irrigation. Elevated proline concentrations were observed during early stress stages (2 days), particularly in NaA and NaA/proline treatments. Proline levels were suppressed after 15 d, indicating an adaptive metabolic response in NaA, NaA/Melatonin, proline as well as on NaA/Proline primed plants. Significant downregulation of enzymatic antioxidants (SOD, CAT and H_2_O_2_) was also observed in all treatments in the initial stages of stress application and were maintained at low levels throughout the duration of the experiment until 29 days. Thereafter, most biochemical markers returned to baseline levels, indicating stabilization of physiological responses under sustained deficit irrigation. The findings highlight the potential of specific priming strategies, particularly those involving Mel, NaA, and NaA/proline to mitigate drought stress, sustain yield, and improve physiological resilience in strawberry cultivation under water deficit conditions.

**Supplementary Information:**

The online version contains supplementary material available at 10.1038/s41598-025-29763-z.

## Introduction

Strawberry (*Fragaria x ananassa*) fruit is receiving continuous attention, mainly due to its appealing texture and its health-promoting properties^1^. Currently, its production has expanded in 76 countries, steadily increasing the economic importance, while China is the largest strawberry producer followed by USA and European Union (EU). Within the EU, Spain, Poland and Germany lead in production volumes followed by the United Kingdom, Italy, France and Greece^2^.

Adaptability of the species has been one of the main reasons for its global expansion. Despite multiple breeding programs, crop management techniques and new systems having been employed by the industry, strawberry production is still challenged by various environmental factors which have driven researchers to develop new strategies that aim to meet the growing demand for premium strawberry fruits^1^.

Reproductive and vegetative system of strawberry plants exhibit sensitivity not only to elevated temperatures and photoperiod, but also multiple agronomic stresses like mineral nutrition, high soil salinity and limited water availability^3–5^. Water limitation is one of the leading environmental stresses that lead to decreased crop growth and production, while prolonged stress exposure has a significant impact on crop physiology and subsequently on plant yield performance^6^. Such events are common in Mediterranean ‘hot-spot’ zones like Cyprus that suffers both from adverse climate conditions and water scarcity.

Strawberry is a water-demanding crop, since productivity highly depends on the volume of irrigation water received. However, this presents an issue in areas characterized by low summer rainfall and restricted water sources for irrigation which can pose significant challenges to strawberry cultivation^7^. The emergence of new and effective strategies to alleviate abiotic stress is crucial for ensuring sustainable agriculture and food security. Recently, the application of chemical compounds to plants which improve their tolerance against such stresses has been gaining more attention. These compounds confer stress tolerance by triggerring molecular and physiological cascades that offer a protective effect to the plants. This phenomenon is commonly referred to as chemical priming^8–10^. Various natural compounds have the ability to function as priming agents against a variety of abiotic stresses, such as amino acids like proline, hormones such as salicylic acid, reactive oxygen-nitrogen-sulfur species (RONSS), polyamines, melatonin, etc., with various reports demonstrating protective effects including strawberry as a target crop^4,11^. Notably, synthetic chemistry can also be used to create effective priming agents, including fungicidal compounds like strobilurins and NOSH-aspirin (NBS-1120), a new hybrid that releases nitric oxide (NO) and hydrogen sulphide (H_2_S)^8,9,12–14^. Proline is naturally occurring in high concentrations in plant tissues under abiotic stress conditions, being involved in signaling, osmotic adjustment, ROS scavenging, cellular pH and redox balance maintenance^15,16^. It has been demonstrated in multiple studies that exogenous application of proline can have a protective effect in plant tissues against abiotic stresses such as drought^16^. Noteworthy, several recent studies have indicated that exogenous application of melatonin increase proline content and overall enhance plant performance under stress^17^. showed that application of melatonin under conditions of water limitation, affected *PIP* transcript levels, enzyme activity, ABA and amino acid content, which all had a positive effect of hot pepper growth. Mechanistic action of melatonin pre-treatment might be linked with the overexpression of genes related to cell division, photosynthesis, carbohydrate metabolism, fatty acid biosynthesis and ascorbate metabolism as demonstrated in soybeans^18^. The use of chemical (natural or synthetic) agents to prime plants against abiotic stresses, is a promising and sustainable approach to crop management. Furthermore, the use of nanocarriers (including biopolymers such as sodium alginate) as smart delivery vectors for chemical priming agents towards improved plant performance is attracting increasing attention^19^. However, additional work is needed to optimize these strategies and validate their effectiveness under field and commercial conditions^14^.

This study aimed to investigate the effect of different PAs in a commercial strawberry plantation under deficit irrigation conditions. The objective was to investigate whether priming can enhance or maintain productivity of strawberries under limited water availability, which is a common issue in the Mediterranean zone. We hypothesized that the exogenous pre-flowering application of the PAs in strawberry plants will result in improved plant performance under limited water availability, leading to improved yield when compared to hydroprimed plants.

## Materials and methods

### Plant material and experimental design

The experiment was conducted from October 2023 to February 2024 in a commercial strawberry field located in Astromeritis village, Nicosia district (160 m (520 ft) above sea level, latitude: 35°8′0″N, longitude: 33°2′0″E). The region has a classic Mediterranean climate, with moderate winters and hot, dry summers. This study used fresh rooted strawberry cv. ‘Red Sayma 1075’ plants were provided by Berryplasma (Varda Ilias, Greece) as fresh tray plants that were subsequently transplanted into 6.5 L pots under net followed a randomized complete block design (RCBD), with five blocks serving as biological replicates. Treatments were randomized within each block and strawberry plants were irrigated with 50% of standard irrigation after the last priming application to simulate conditions of deficit irrigation. In total, ten plants were used per treatment.

## Treatment applications and sampling procedure

The following treatments were allocated in each block: hydroprimed (deionized H_2_O), 100 µM melatonin (Mel), 0.1% w/v sodium alginate (NaA), 0.1% w/v NaA/100 µM melatonin (Mel), 2 mM proline, and 0.1% w/v NaA/2 mM proline. To ensure that the priming agents bind to the leaf surface, each solution contained 0.1% v/v Tween 20 surfactant. Throughout the experiment, three priming applications were performed. The inaugural application aimed to combat transplantation stress, while the other two were performed at different developmental stages. In particular, ten plantlets received 15 ml of the corresponding treatment at the root zone, 2 d before transplantation into 6.5 L pots which contained peat: perlite substrate at a ratio of 2:1. The foliar treatments were performed at 8 d and 15 d after transplantation. To ensure that adequate quantity of PAs was delivered to the plants, foliar applications were performed until complete runoff. Deficit irrigation was implemented 4 d after the last priming application.

Two fully expanded strawberry leaves were collected per biological replication from each treatment at 2, 15 and 29 d after imposition of deficit irrigation. The collected material was initially frozen in dry ice in the field until stored at −80 °C. Such material was analysed for cellular damage indicators, enzymatic activity assays, photosynthetic pigments and antioxidant activity, as described in the following sections.

## Yield performance

Strawberry plants used for yield measurements were randomly assigned and used throughout the experiment. The number and weight of each strawberry, as well as the total weight of strawberries produced per plant, were recorded. For each treatment, 5 strawberry plants were used to measure yield. The cumulative yield per plant was calculated by adding the weight of all strawberries collected from the same plant during the harvesting period according to^20^. In order to effectively capture the effects of priming, three early harvests of commercially mature strawberries were performed (November 23, November 28, December 5) over the experimental period. Such early harvested fruits have considerably high market value due to limited supply over the period being produced.

## Quantification of endogenous melatonin

Melatonin extraction assay was performed according to the manufacturer’s instructions using an Melatonin ELISA Kit (Enzo Life Sciences, Farmingdale, NY, USA). In summary, leaf samples of 0.5 g weight were finely ground into powder using liquid nitrogen and homogenized in 125 µL of 1× stabilizer solution from the kit. Subsequently, 750 µL of cold ethyl acetate was added, and the mixture was subjected to vortexing. After 5 min incubation on ice, the solution was centrifuged at 1000 × g at 4 °C for 10 min. The resulting organic layer was transferred to a new glass tube and allowed to dry overnight. The resulting pellet was reconstituted in 200 µL of 1 × stabilizer for melatonin quantification following the manufacturer’s recommended protocols.

## Lipid peroxidation quantification

The extent of lipid peroxidation was assessed by quantifying malondialdehyde (MDA) content in leaf samples through the thiobarbituric acid (TBA) reaction method^21^. The MDA content was measured at 532 nm and 600 nm (TECAN, Infinite 200^®^ PRO) and estimated with the help of the Lambert-Beer law, with an extinction coefficient of 155 mM^− 1^cm^− 1^ and expressed as nmol g^− 1^ fresh weight (FW).

### Reactive oxygen species quantification

Hydrogen peroxide (H_2_O_2_) content was quantified using a spectrophotometric method, which relies on the oxidation of iodide (I^− 1^) to iodine (I) after H_2_O_2_ reaction with potassium iodide (KI), as described by^22^. The H_2_O_2_ content was measured at 390 nm (TECAN, Infinite 200^®^ PRO) and a standard curve of known concentrations (µmol H_2_O_2_ g^− 1^ FW) was used for the estimation.

## Proline quantification

The ninhydrin reaction was used to measure the levels of free proline in leaf samples, as proposed by^23^. Proline concentration was determined by measuring at 520 nm (TECAN, Infinite 200^®^ PRO), estimated from a standard curve, and expressed on a fresh weight basis (µmol proline g^− 1^ FW)^21^.

## Enzymatic activity assays

To extract superoxide dismutase (SOD) and catalase (CAT), leaf samples (0.1 g) were homogenized in ice-cold extraction buffer (100 mmol L^− 1^ phosphate buffer pH = 7.5, 0.5 mmol L^− 1^ EDTA, 1 mmol L^− 1^ PMSF) with the use of a mortar and pestle. Each homogenate was centrifuged (16000 × *g*, 4 °C for 20 min, Eppendorf Centrifuge 5415 R) and total supernatant was used for enzymatic activity assay. Total SOD activity was assessed by evaluating its capacity to inhibit the photochemical reduction of nitro blue tetrazolium chloride (NBT) as described by^24^, with minor alterations. The reaction mixture contained 50 mmol L^− 1^ phosphate buffer (pH = 7.8), 13 mmol L^− 1^ methionine, 75 µmol L^− 1^ NBT, 0.1 mmol L^− 1^ EDTA, 2 µmol L^− 1^ riboflavin and 50 µL of enzyme extract in a final assay volume of 1.5 mL. The photoreduction of NBT was measured spectrophotometrically at 560 nm with the reduction being inversely proportional to SOD activity^25^. The reaction mixture without enzyme exhibited the maximum color due to maximum reduction of NBT and was used as the control. The blank solution exhibited the same complete reaction mixture but it was kept in the dark. One unit of SOD activity (U) was defined as the amount of enzyme needed to achieve 50% inhibition of the NBT photoreduction rate. The results were expressed as specific activity units mg^− 1^ protein. Catalase (CAT) activity was measured according to^26^ with slight modifications. The reaction mixture consisted of 50 mmol L^− 1^ potassium phosphate buffer (pH = 7), 10 mmol L^− 1^ H_2_O_2_ and 200 µL enzyme extract to a final volume of 1.5 mL. The rate of H_2_O_2_ disappearance (extinction coefficient 39.4 mM^− 1^ cm^− 1^) was assessed at 240 nm during 1 min and results were expressed as specific activity units mg^− 1^ protein^27^. Protein content of all samples was calculated using the Bradford method^28^.

### Photosynthetic pigment analysis

To obtain pigment extractions, 5 leaf disks (approximately 25 mg/1.5 ml) per sample were incubated in 5 ml dimethyl sulfoxide **(**DMSO) for 30 min at 65 °C and absorbance of samples was measured at 661, 643, 470, and 534 nm, respectively^29^. Chlorophylls (Chla, Chlb and total) and carotenoids concentrations were defined using the equations described by^30^, while anthocyanins concentration was determined using the equations described by^31^.

### Total antioxidant capacity

The Ferric Reducing Antioxidant Power (FRAP) and the phosphomolybdenum method were assesed. Two mL of methanol was added to 0.05 g of strawberry leaves, vortexed and placed at 4 °C for 24 h. Subsequently, the mixtures were centrifuged for 10 min at 16,000 × g at 4 °C (Eppendorf Centrifuge 5415 R), and the supernatant was stored in sealed vials at −20 °C for further analysis.

For the FRAP method, 1.98 mL of freshly prepared FRAP solution [0.3 mol L^− 1^ acetate buffer (pH = 3.6) containing 10 mmol L^− 1^ 2,4,6- tripyridyl-1,3,5-triazine (TPTZ) and 40 mmol L^− 1^ FeCl310H2O] was mixed with 10 µL of methanol extract. The mixture was incubated at 37 °C for 4 min and the absorbance was measured at 593 nm (TECAN, Infinite 200^®^ PRO). A standard curve was constructed and results were expressed as µmol ascorbic acid g^− 1^ FW^32^.

For the phosphomolybdenum method, 50 µL methanol extract was mixed with 1 mL of reagent solution (0.6 mol L^− 1^ sulfuric acid, 28 mmol L^− 1^ sodium phosphate, and 4 mmol L^− 1^ ammonium molybdate) and the tubes were incubated at 95 °C for 90 min. The absorbance of the solution was measured at 695 nm against a blank sample, and the total antioxidant capacity was expressed as µmol L-ascorbic acid g^− 1^ FW^32^.

### Statistical analysis

Statistical analysis and figures were performed using GraphPad Prism version 10.4.1 (GraphPad Software, San Diego, CA) and comparisons between priming agent application at three timepoints after deficit irrigation (2 d, 15 d and 29 d) were evaluated by using the two-way ANOVA followed Tukey-HSD post hoc test (*P* ≤ 0.05). Principal component analysis (PCA) and heatmap were created using ClustVis 2.0 according to^33^ and the data were normalized to hydroprimed. Euclidean distance was used as the clustering distance metric.

## Results

### Strawberry early production under deficit irrigation

**Fig. 1:**
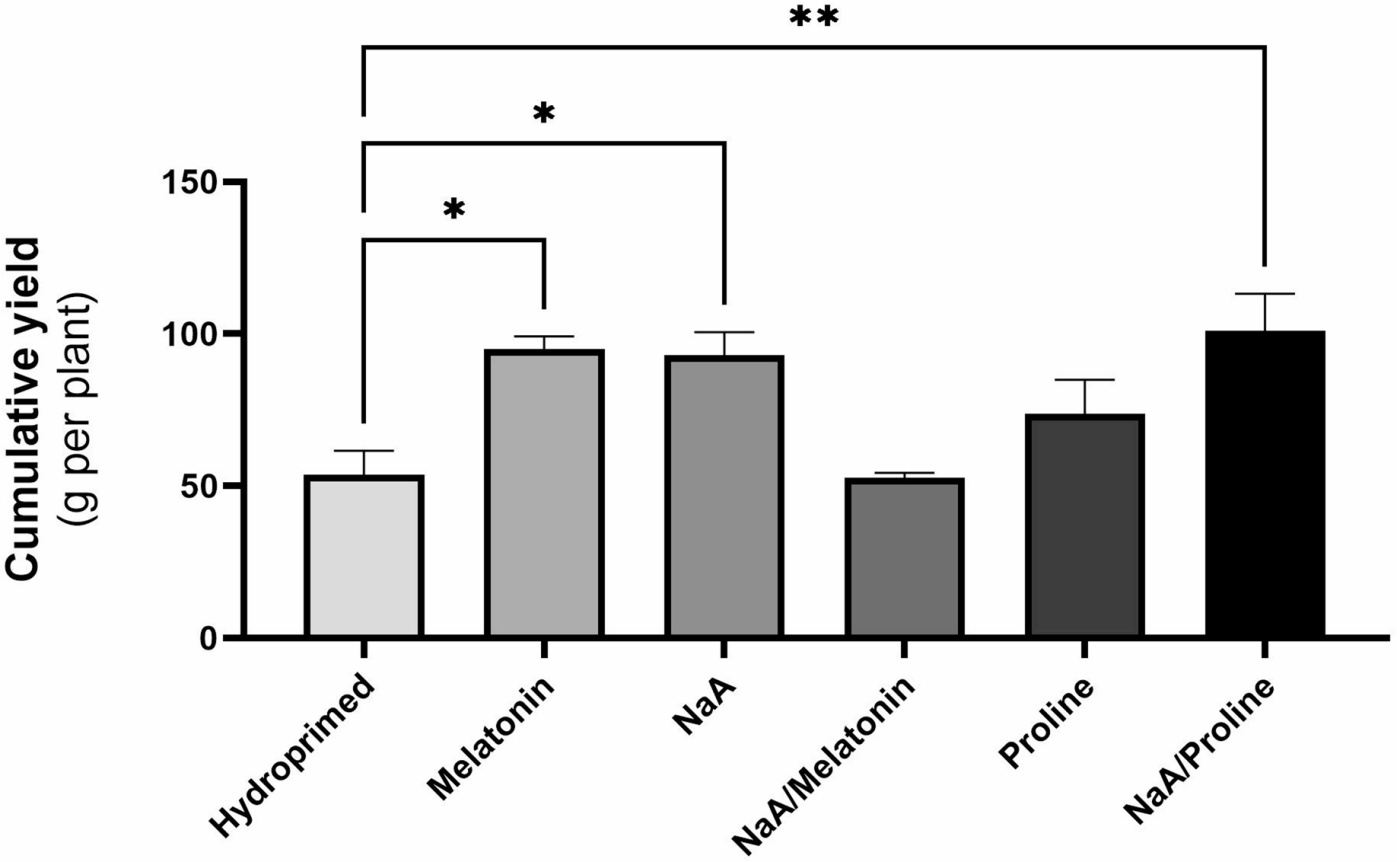
Cumulative yield of strawberry fruits (g per plant) for the total of the three early harvests on plants subjected to the following treatments with priming agents: hydroprimed, melatonin (100 µM), sodium alginate (0.1% w/v, NaA), NaA/Melatonin conjugate (0.1% w/v/100 µM), proline (2 mM), and NaA/Proline conjugate (0.1% w/v/2 mM). *ns = p > 0.05*, * = p ≤ 0.05, ** = p ≤ 0.01, *** = p ≤ 0.001, **** = p ≤ 0.0001.

Priming significantly affected plant productivity under deficit irrigation. Treatments with melatonin (Mel), as well as sodium alginate (NaA) almost doubled the cumulative yield per plant compared with the hydroprimed controls (*p* < 0.05). Similarly, priming with proline and sodium alginate conjugate (NaA/Proline) had the most substantial improvement in cumulative yield (*p* < 0.01) (Fig. 1). Contrarily, plants treated with the NaA/Melatonin conjugate did not lead to increased early yield compared with hydroprimed plants, while proline-treated plants resulted in a slight yet not significant increment of early yield compared with hydroprimed ones. Interestingly, average berry number per plant was significantly increased in Mel-treated plants while average berry weight was similar between treatments (**Supplementary Table 1**).

### Endogenous melatonin content

**Fig. 2:**
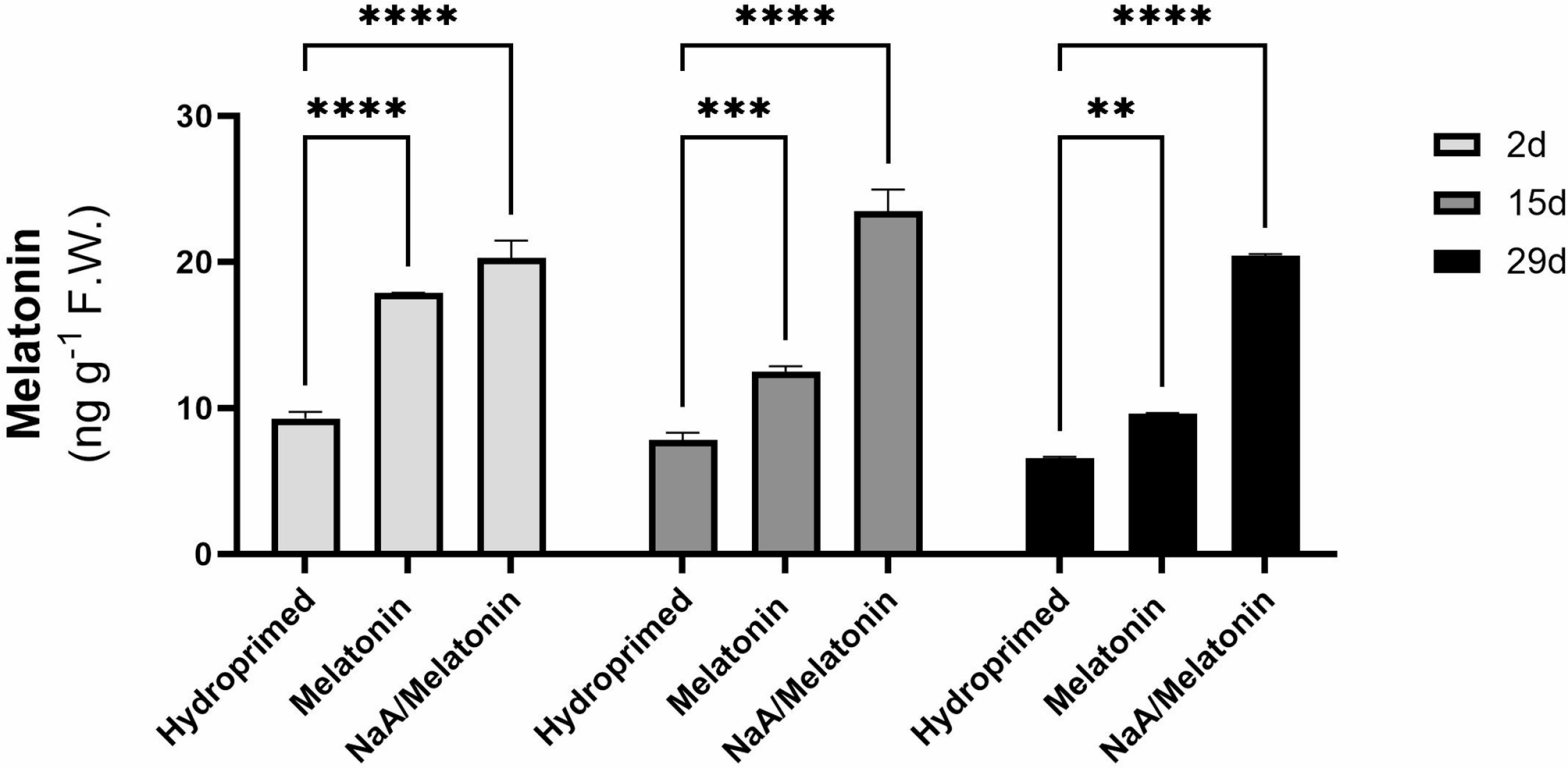
Effect of priming agents on melatonin content in strawberry leaves collected at three timepoints (2 d, 15 d and 29 d after deficit irrigation imposition). Treatments with priming agents are described in Fig. 1. *ns = p > 0.05*, * = p ≤ 0.05, ** = p ≤ 0.01, *** = p ≤ 0.001, **** = p ≤ 0.0001.

Endogenous melatonin (Mel) content was quantified, in order to evaluate the efficiency of absorbance and persistence of Mel in leaf tissue through the exogenous application of Mel. Treatments involving Mel alone or NaA/Mel, had significantly higher endogenous Mel content compared with the hydroprimed controls, validating the successful donation (Fig. 2). Specifically, 2 d after stress implementation, both Mel and NaA/Mel treatments showed significantly higher endogenous Mel content compared with hydroprimed controls (*p* < 0.0001). Two weeks after stress implementation (15 d), Mel content remained higher compared to the hydroprimed in both treatments. Mel treatment had significantly higher endogenous Mel content compared with hydroprimed samples (*p* < 0.01), but not as high as the first time-point (2 d). NaA/Mel treatment maintained the high levels of endogenous Mel content more effectively at 15 d after stress implementation (*p* < 0.001). Similar pattern was observed at 29 d after stress imposition, where NaA/Mel treatment maintained high levels of endogenous Mel content (*p* < 0.001) across all time-points of leaf collection. While priming with Mel alone displayed a significant increase in the endogenous content of Mel compared with hydroprimed samples (*p* < 0.01), there was a decline over the course of the experiment, while NaA/Mel treatment maintained endogenous Mel content (Fig. 2).

### Cellular damage indicators

**Fig. 3:**
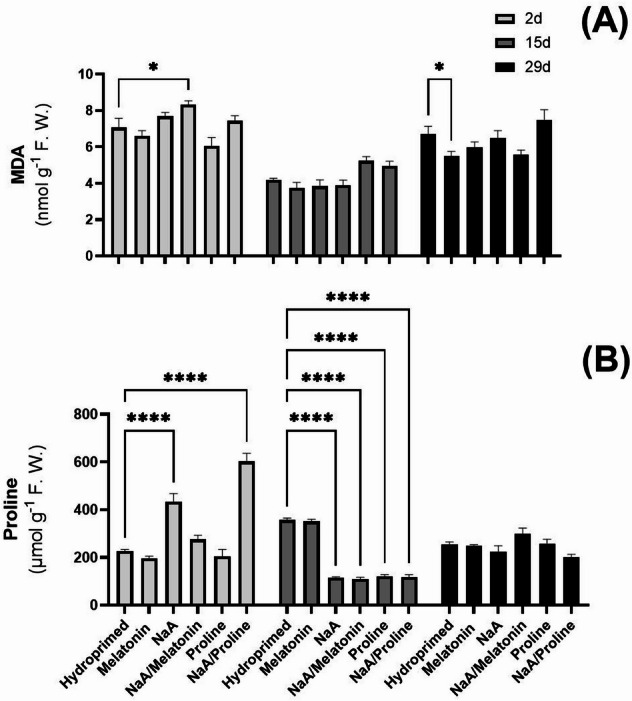
Effect of priming agent application on (A) MDA content and (B) proline content in strawberry leaves harvested at three timepoints (2 d, 15 d and 29 d after deficit irrigation imposition). Treatments with priming agents are described in Fig. 1. *ns = p > 0.05*, * = p ≤ 0.05, ** = p ≤ 0.01, *** = p ≤ 0.001, **** = p ≤ 0.0001.

**Fig. 4:**
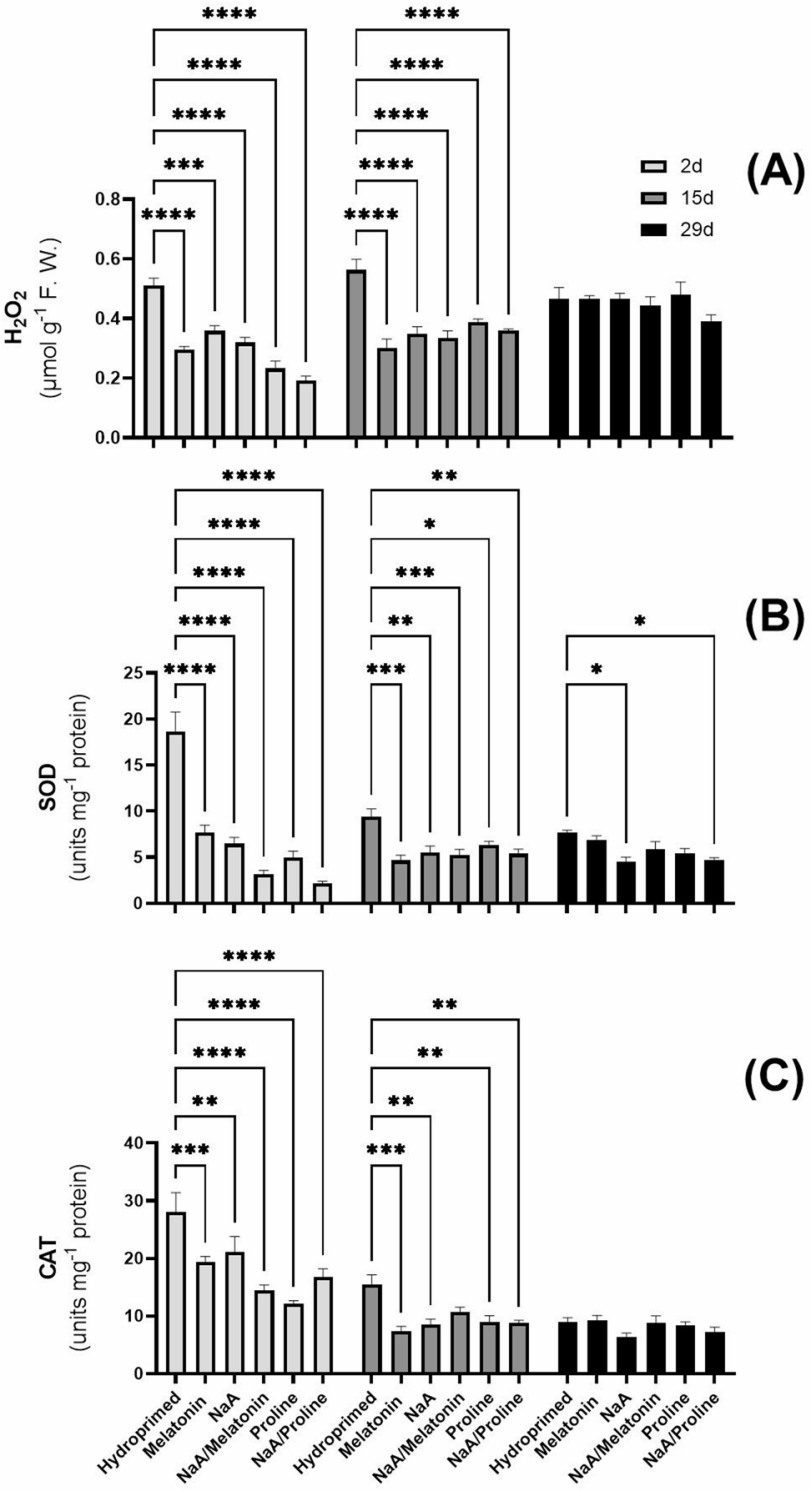
Effect of the application of priming agents on (A) H_2_O_2_ content, (B) SOD enzymatic activity and (C) CAT enzymatic activity in strawberry leaves harvested at three timepoints (2 d, 15 d and 29 d after deficit irrigation imposition). Treatments with priming agents are described in Fig. 1. *ns = p > 0.05*, * = p ≤ 0.05, ** = p ≤ 0.01, *** = p ≤ 0.001, **** = p ≤ 0.0001.

**Fig. 5:**
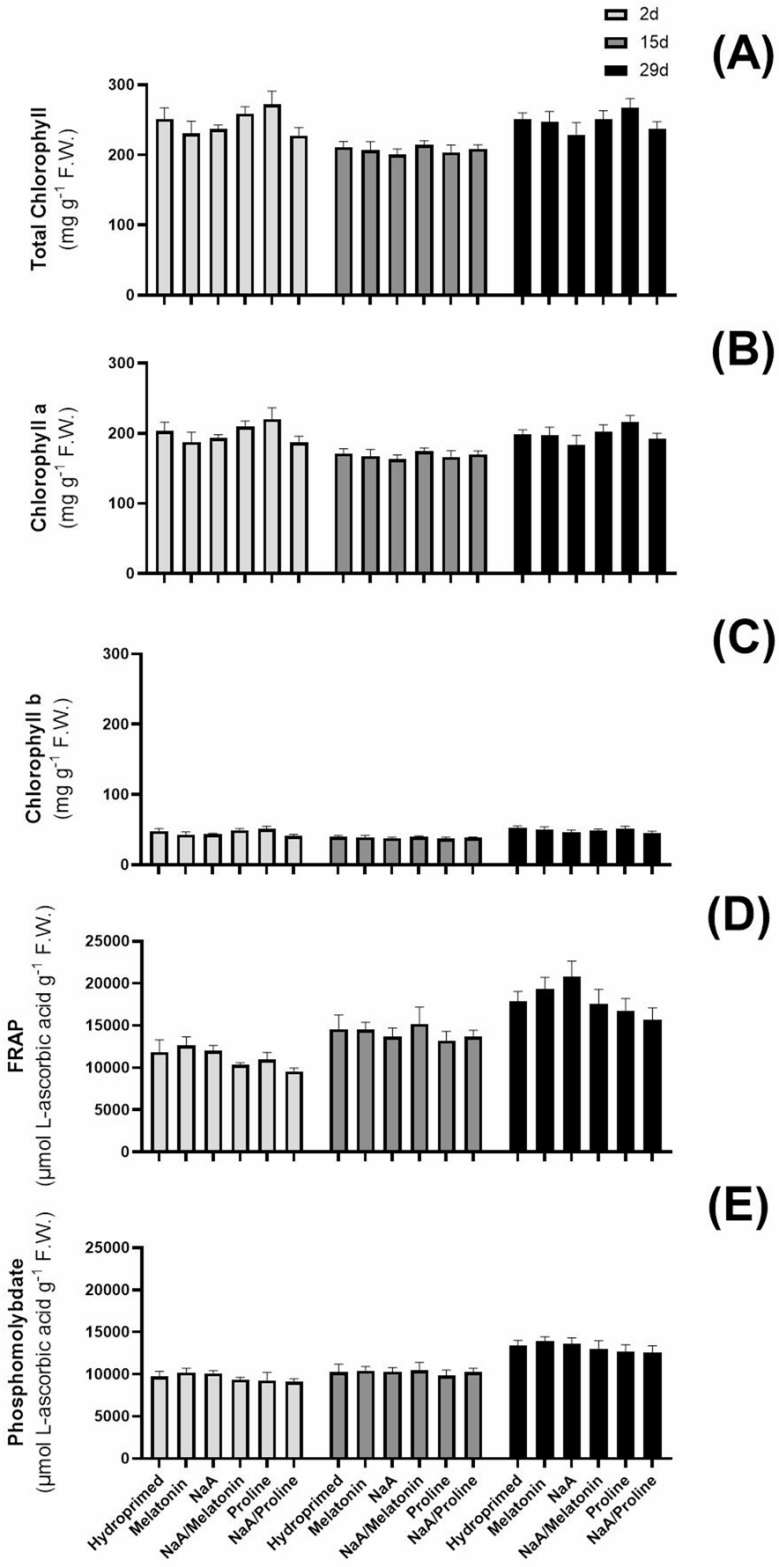
Effect of the application of priming agents on (A) total chlorophylls, (B) chlorophyll a, (C) chlorophyll b, (D) FRAP antioxidant activity and (E) phosphomolybdate antioxidant activity in strawberry leaves harvested at three timepoints (2 d, 15 d and 29 d after deficit irrigation imposition). Treatments with priming agents are described in Fig. 1. *ns = p > 0.05*, * = p ≤ 0.05, ** = p ≤ 0.01, *** = p ≤ 0.001, **** = p ≤ 0.0001.

Major differences between treatments in MDA content were not observed. However, MDA content dropped significantly after 15 d of deficit irrigation and increased again by 29 d (Fig. 3A). Proline content exhibited time-dependent variability under deficit irrigation. Two days after stress implementation, priming with NaA and its conjugate with proline (NaA/Pro) significantly increased endogenous proline content compared with hydroprimed controls (*p* < 0.0001) (Fig. 3B). Fifteen days post-stress, all priming applications except Mel treatment, lowered proline content dramatically (*p* < 0.0001) (Fig. 3B). At 29 d, priming did not influence endogenous proline content significantly (Fig. 3B). H_2_O_2_ content was significantly decreased in all priming applications at 15 d post-stress (*p* < 0.0001) (Fig. 4A). After 2 d, proline as well as NaA/Proline-treated plants displayed the lowest H_2_O_2_ contents compared with hydroprimed controls. Although H_2_O_2_ content was slightly increased in all priming applications after 15 d of deficit irrigation, they maintained significantly lower levels compared with hydroprimed samples (*p* < 0.0001) (Fig. 4A). At 29 d, all plants exhibited similar H_2_O_2_ levels independent of the treatment (Fig. 4A). Enzymatic activity of SOD and CAT displayed similar trends in terms of content (Fig. 4B and C). All priming applications were highly effective at lowering the level of SOD, with the NaA/Proline conjugate having the lowest SOD activity (*p* < 0.0001) 2 d post-stress implementation. Over time, SOD activity decreased across all treatments, maintaining significantly lower levels compared with hydroprimed samples. At 29 d, SOD activity was maintained relatively stable across all treatments with the exception of NaA and NaA/Proline treatments, which had significantly lower SOD activity compared with hydroprimed plants (*p* < 0.05) (Fig. 4B). CAT enzymatic activity followed a similar trend with all priming treatments exhibiting a significant decrease compared with hydroprimed plants at 2 d and 15 d post-stress implementation (Fig. 4C). Similar to SOD activity, CAT enzymatic activity progressively decreased across time. Despite the gradual decrease in enzymatic activity, at 15 d of deficit irrigation, almost all treatments except for NaA/Melatonin continued to have significantly lower enzymatic activity of CAT compared with hydroprimed plants (Fig. 4C). Mel treatment showed the lowest and most significant decrease in CAT activity compared with hydroprimed samples (*p* < 0.001). At 29 d of deficit irrigation, CAT activity remained stable in all treatments (Fig. 4C). Photosynthetic pigment content and overall antioxidant capacity did not exhibit any differences across treatments or time-points of leaf collection (Fig. 5A-E).

### Principal component analysis (PCA)

**Fig. 6:**
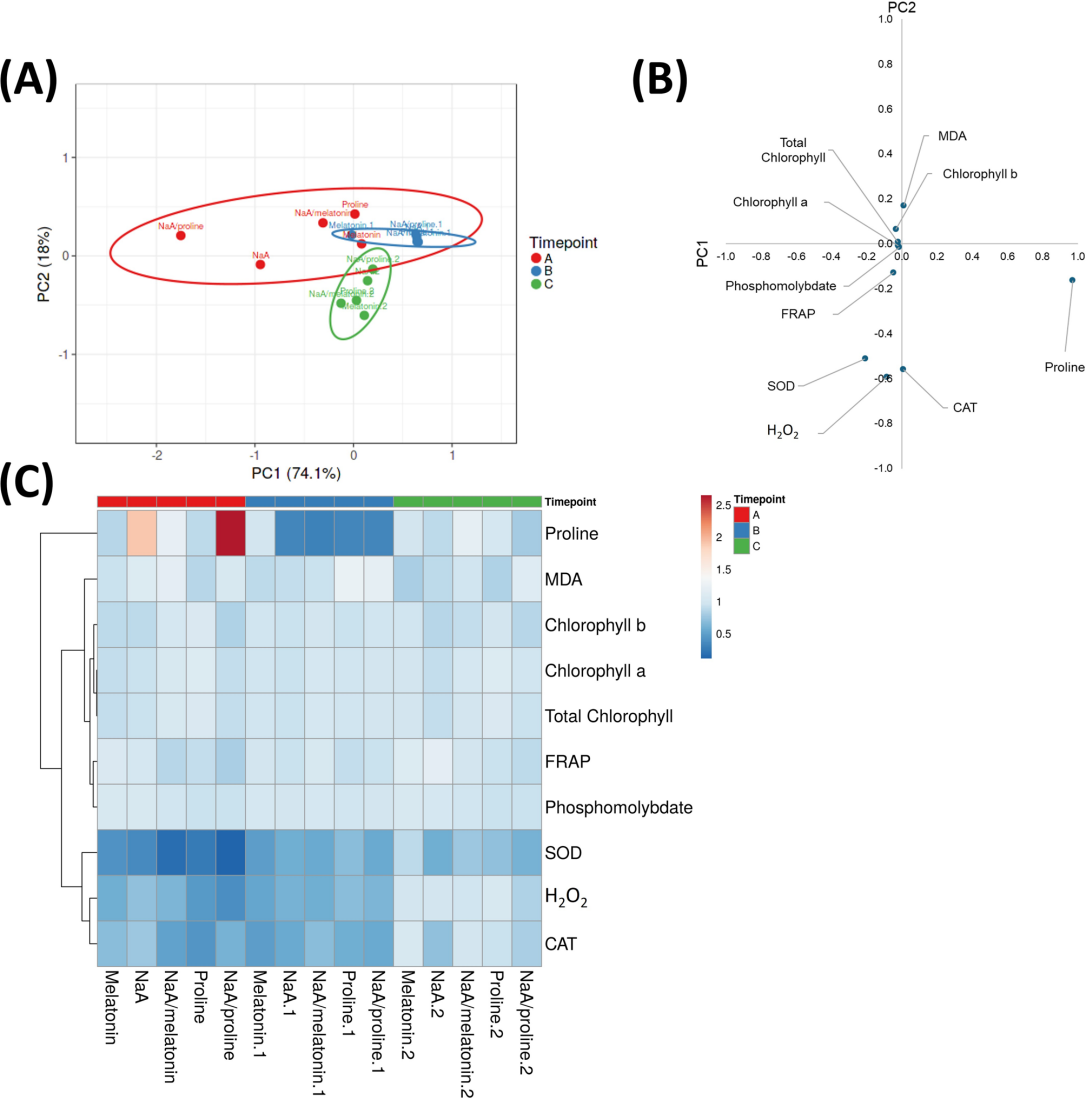
(**A**) Principal component analysis (PCA), (**B**) xy-scatter plot graph of biochemical and enzymatic assays and (**C**) heatmap representing fold changes of deficit irrigation and priming agent application on biochemical and enzymatic assays in strawberry leaves harvested at three timepoints (2 d, 15 d and 29 d after deficit irrigation imposition). Treatments with priming agents are described in Fig. 1. Data were normalized vs. hydroprimed samples. The normilized data are available in **Supplementary Tables 2 and 3**. ns = p > 0.05, * = p ≤ 0.05, ** = p ≤ 0.01, *** = p ≤ 0.001, **** = p ≤ 0.0001.

The first two principal components explain 92.1% of the total variance (PC1 = 74.1%, PC2 = 18% respectively). Based on PCA plot, 2 d (Timepoint A) after deficit irrigation, treatments exhibited the highest dispersion compared with the other timepoints. Specifically, there was a strong separation of NaA/Proline and NaA treatments on Timepoint A of leaf collection (Red cluster), which reflects differences in proline accumulation (Fig. 6A and B; **Supplementary Table 2**). PC1 explains the majority of variation observed in the data with proline content to have a strong positive score on PC1 (0.972) while other variables had very small or negative scores suggesting that PC1 is strongly associated with proline content (Fig. 6A and B; **Supplementary Table 2**). Principal component 2 (PC2), captures the maximum remaining variance in the data. In PC2, parameters of H_2_O_2_ (−0.592), CAT (−0.559) and SOD (−0.511) showed the most negative scores, suggesting that PC2 is driven by antioxidant enzymes (SOD & CAT) and oxidative stress (H_2_O_2_) (Fig. 6A and B; **Supplementary Table 2**). Timepoint B (15 d) exhibited intermediate separation (Blue cluster), whereas in Timepoint C (29d; Green cluster) all treatments were clustered more tightly together, indicating similar profiles across treatments (Fig. 6A and B; **Supplementary Table 2**).

### Heatmap of biochemical markers

Heatmap of normalized fold-changes to the hydroprimed samples was used to visualize the physiological responses of strawberry plants to deficit irrigation at different time-points and treatments, across multiple biochemical assays (Fig. 6C). Notable increases in proline content were observed in early days of deficit irrigation (Timepoint A), with NaA (Fold-change < 2) and NaA/Proline (Fold-change > 2) treatments having the most significant effects across all treatments (Fig. 6C; **Supplementary Table 3**). Substantial downregulation of enzymatic antioxidants was also observed in all treatments during the initial stages of stress implementation and were maintained at low levels for the duration of the experiment until Timepoint C (29 d after stress initiation) (Fig. 6C; **Supplementary Table 3**). At Timepoint B, proline content displayed strong down-regulation compared with hydroprimed samples in NaA, A/Melatonin, proline as well as NaA/Proline-primed plants. After 29 d of deficit irrigation, the majority of biochemical markers returned to values closer to the baseline (Fold-change ~ 1), with a stable profile across treatments (Fig. 6C; **Supplementary Table 3**).

## Discussion

### The effect of priming application on yield performance

**Fig. 7:**
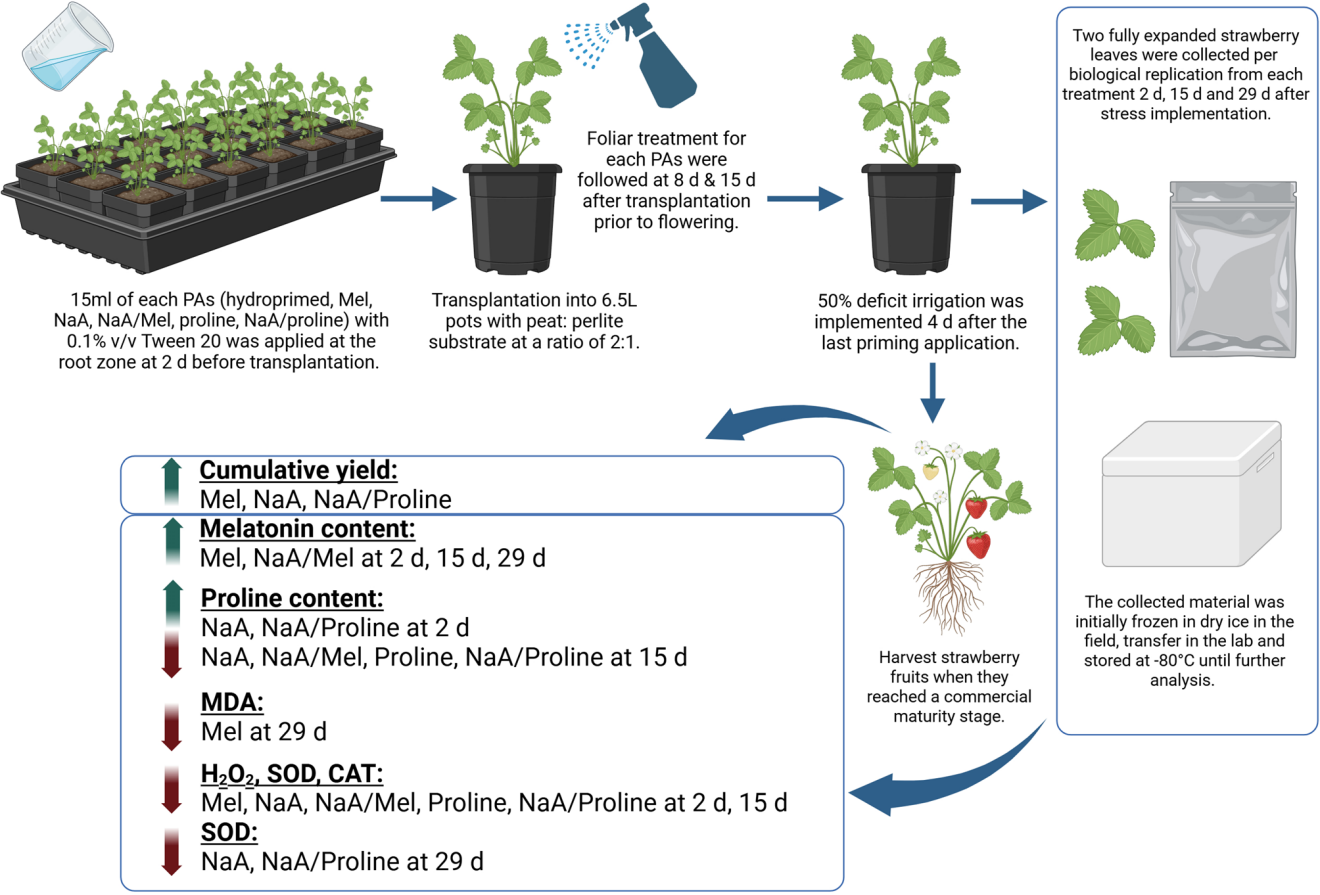
Model summarizing methodological approach and key findings of the study. Created in BioRender. Georgiadou, E. (2025) https://BioRender.com/k4wqbw0.

The current study highlights the effectiveness of proline and Mel, as well as their conjugates with NaA, as priming agents towards enhanced strawberry production under deficit irrigation conditions. Evidence presented herein clearly suggests that exogenous application of specific priming agents, either directly or through smart delivery with the combined use of biopolymers, can mitigate negative effects of limited water availability in strawberry plants by improving biochemical and antioxidant responses (model of methodology and key findings is presented in Fig. 7). These observations are consistent with the increasing number of reports promoting the application of priming agents as an eco-friendly tool to counteract abiotic stress conditions in crops^10^. The most promising result of our study was the significant increase in cumulative yield during the early harvests in Mel-, NaA-, and NaA/Proline-treated plants compared with hydroprimed controls. The NaA/Proline treatment demonstrated optimal effects, with cumulative yield almost doubling, which highlights the synergistic promise of the integration of osmoprotectants with biopolymers such as sodium alginate. Interestingly, average berry number per plant was significantly increased only in Mel-treated plants while average berry weight was similar between treatments, suggesting that observed increases in yield could be at least partially attributed to promotion of fruit setting rather than increased average weight. These findings are supported by earlier reports, where they examined the effect of the weekly application of Mel was examined in strawberries grown under different salinity conditions^34^. Yield benefits through Mel application were also reported in other non-fruit crops like maize, in which Mel enhanced plant growth by regulating photosynthetic efficiency and osmolyte biosynthesis in maize seedlings grown under drought stress^35^. Similarly, priming with proline has also been reported to increase strawberry yield under deficit irrigation regimes^36^.

The long-sustained yield performance of primed plants indicates that chemical priming not only alleviates immediate stress, but also induces long-term physiological stability. However, to what proportion the PAs can increase yield should be examined in commercial setups with a high number of plants.

### Melatonin-related stress adaptation

Mel-primed as well as NaA/Mel-primed plants illustrated elevated and sustained endogenous Mel content across an extended period of deficit irrigation confirming successful donation of the priming agent, with NaA/Mel treatment to be the most effective under prolonged periods of stress. Such findings are in agreement with the work of^37^, who showed that Mel-primed alfalfa plants exhibit higher endogenous Mel content correlated with improved drought stress tolerance, attributed to the known role of Mel in the regulation of antioxidant defenses and stress signaling pathways^38^. Notably, NaA/Mel-treated plants maintained higher Mel concentrations over time, which could be attributed to the controlled-release properties of NaA^39^. The comparative decline in Mel content in plants treated with Mel alone during the course of the experiment suggests that conjugation with NaA could optimize the delivery and persistence of priming agents under real-life agricultural (i.e. field or glasshouse) conditions.

#### Proline accumulation and osmotic adjustment

The sharp increase in proline content at 2 d post-stress initiation in NaA and NaA/Proline-treated plants suggests an early adaptation to water deficit. Proline is an extensively studied osmoprotectant that stabilizes cellular structures and scavenges ROS under stress conditions^15^. The subsequent lowering in proline content in NaA and NaA/Proline-treated plants at 15 d suggests that plants may have become acclimated, transitioning from acute stress response to restored metabolic balance. In contrast, Mel-primed plants maintained relatively stable proline levels over the duration of the experiment, indicating a more sustained but moderate osmotic adjustment unlike the one observed in melatonin-treated peanut seedlings under drought stress where proline levels increased significantly^40^. Current proline dynamics are consistent with findings by^41^ for drought-stressed strawberries, where proline accumulation correlated strongly with the duration and severity of the stress. We hereby postulate for fist time that the optimal performance recorded in NaA/Proline-treated plants suggests that NaA could potentially be enhancing the bioavailability of proline or prolonging its action, which warrants further research into carrier systems for priming agents.

### Regulation of oxidative stress responses

All priming treatments resulted in significant lowering in H₂O₂ content, as well as in down-regulated SOD and CAT enzymatic activities in comparison with hydroprimed samples in the first 15 d post-stress initiation, indicating successful mitigation of oxidative stress induced by deficit irrigation by proline as well as Mel and their conjugates with NaA. These results are in accordance with previous reports, highlighting the antioxidant-modulating properties of melatonin and proline through enhanced ROS scavenging and stress signaling^38,42^. Interestingly, these parameters (as well as MDA content) returned to control levels across all treatments at 29 d, indicating a recovery phase and restored cellular homeostasis that support the notion that priming accelerates stress acclimation^9^. Furthermore, the fluctuation observed in MDA content in particular could be indicative of the biphasic nature of the plant’s response, whereby early stress/priming phase would lead to rapid and efficient ROS detoxification linked with lower MDA content, while prolonged stress exposure might lead to oxidative damage accumulation. to Interestingly, no significant differences were observed among priming treatments in total antioxidant capacity or photosynthetic pigment content, suggesting that the protective effect of chemical priming operates mainly via dynamic enzymatic antioxidant and osmolyte-mediated adjustments, rather than by enhancing photosynthetic pigment content or non-specific antioxidant capacity. Such an example is the work of^43^, who showed that melatonin treatment in agronomic crops as alfalfa plants resulted in improved performance under drought stress conditions, linked with increased photosynthetic pigment content and enhanced net photosynthetic rate, stomatal conductance, transpiration rate, and intercellular CO_2_ concentration. Besides agronomic crops, further studies on the efficacy of Mel treatment on other fruit crops are needed to validate our research hypothesis.

Our findings were further supported by multivariate statistical analyses, which clearly discriminated treatment groups at early stress stages (with 2 d in particular), with SOD, CAT and proline being the primary discriminating variables. Convergence of biochemical profiles across treatments at the final sampling time (29 d) implies that physiological adaptation was ultimately achieved within the duration of the experiment.

## Conclusions

The present study provides comprehensive insights into the impact of selected priming agents on a wide array of morphophysiological and biochemical parameters, following treatment of the strawberry cultivar ‘Red Sayma 1075’ grown under 50% deficit irrigation. The findings highlight the promising role of Mel, NaA, as well as the conjugated form of Pro with NaA as a sustainable strategy to enhance strawberry fruit productivity under water-limited conditions, as evidenced by the significant increase in cumulative yield under moderate drought stress conditions following these treatments with optimal yield being obtained by the NaA/Pro treatment. Such improvements in performance under stress could be attributed, at least partly, to the recorded regulation of the antioxidant apparatus along with osmotic adjustment primarily by Mel, NaA, and NaA/Pro, with the optimal performance being recorded in NaA/Pro-treated plants which suggests that synergistic effects in their modes of action may well be taking place. It is interesting to note that conjugated forms of NaA with priming agents were recently shown to also have beneficial effects in bioactive compound profiles in strawberry fruit from plants grown under control conditions^44^, highlighting an additional benefit of such approaches. Nevertheless, some important limitations should be noted. The absence of significant differences in photosynthetic pigments and overall antioxidant capacity indicates that the advantages of priming could be more pronounced under more severe stress conditions rather than constitutive characteristics, while this could also be attributed to genotype-dependent responses of the specific cultivar. Further studies are therefore needed to elucidate the underlying *modus operandi* of these priming agents, potentially through the application of state-of-the-art omics platforms, while evaluation of more strawberry cultivars and abiotic stress conditions would enhance the translational applicability of these results.

## Supplementary Information

Below is the link to the electronic supplementary material.

Supplementary Material 1

## Data Availability

Data are provided within the manuscript or supplementary information files. The datasets used and/or analyzed during the current study are available from the corresponding author (Dr. Vasileios Fotopoulos; Email: [vassilis.fotopoulos@cut.ac.cy](mailto: vassilis.fotopoulos@cut.ac.cy); Tel: +357-25002418) upon reasonable request.
