# Supplementary material for: Chemical priming of strawberry plants under deficit irrigation enhances yield efficiency and physiological resilience
Source: Sci Rep. 2025 Dec 1;16:373. doi: 10.1038/s41598-025-29763-z (PMC12769752; doi:10.1038/s41598-025-29763-z)
Supplement: Supplementary file 1 — Supplementary Material 1 [file 41598_2025_29763_MOESM1_ESM.docx]

**Supplementary material**

**Supplementary Table 1.** Average berry number per plant and average berry weight for the total of the three early harvests on plants subjected to the following treatments with priming agents: hydroprimed, melatonin (100 μM), sodium alginate (0.1% w/v, NaA), NaA/Melatonin conjugate (0.1% w/v / 100 μM), proline (2 mM), and NaA/Proline conjugate (0.1% w/v / 2 mM).

|  | Average berry number per plant | Average berry weight |
| --- | --- | --- |
| Hydroprimed | 2.60 ± 0.24 b | 24.53 ± 1.05 a |
| Melatonin | 3.80 ± 0.20 a | 23.70 ± 0.72 a |
| NaA | 2.80 ± 0.73 b | 22.55 ± 5.65 a |
| NaA/Melatonin | 2.20 ± 0.37 b | 24.40 ± 1.60 a |
| Proline | 2.60 ± 0.75 b | 19.88 ± 5.33 a |
| NaA/Proline | 3.20 ± 0.20 b | 28.08 ± 3.40 a |

**Supplementary Table 2.** Principal component 1 (PC 1) and principal component 2 (PC 2) demonstrating which biochemical and enzymatic assays are important in strawberry leaves collected at three timepoints after deficit irrigation and priming agent application. Data were normalized vs hydroprimed samples.

|  | **PC1** | **PC2** |
| --- | --- | --- |
| **MDA** | 0.012 | 0.171 |
| **Proline** | 0.972 | -0.162 |
| **H_2_O_2_** | -0.085 | -0.592 |
| **SOD** | -0.208 | -0.511 |
| **CAT** | 0.009 | -0.559 |
| **Total Chlorophyll** | -0.022 | 0.008 |
| **Chlorophyll a** | -0.020 | -0.008 |
| **Chlorophyll b** | -0.034 | 0.065 |
| **FRAP** | -0.046 | -0.127 |
| **Phosphomolybdate** | -0.014 | -0.015 |

**Supplementary Table 3.** Fold changes of deficit irrigation and priming agent application on biochemical and enzymatic assays in strawberry leaves collected at three timepoints after deficit irrigation and priming agent application. The data were normalized vs hydroprimed samples. Timepoint A: 2 days (d) after stress implementation, Timepoint B: 15 days (d) after stress implementation, Timepoint C: 29 days (d) after stress implementation. ns = p > 0.05, * = p≤0.05, ** = p≤0.01, *** = p≤0.001, **** = p≤0.0001.

|  | **Melatonin** | | | **NaA** | | | **NaA/Melatonin** | | | **Proline** | | | **NaA/Proline** | | |
| --- | --- | --- | --- | --- | --- | --- | --- | --- | --- | --- | --- | --- | --- | --- | --- |
|  | **A** | **B** | **C** | **A** | **B** | **C** | **A** | **B** | **C** | **A** | **B** | **C** | **A** | **B** | **C** |
| **MDA** | 0.935 | 0.895 | 0.818 | 1.088 | 0.917 | 0.887 | 1.178 | 0.931 | 0.964 | 0.859 | 1.249 | 0.829 | 1.052 | 1.184 | 1.114 |
| **Proline** | 0.862 | 0.984 | 0.979 | 1.912 | 0.325 | 0.878 | 1.215 | 0.310 | 1.182 | 0.899 | 0.341 | 1.020 | 2.652 | 0.333 | 0.796 |
| **H_2_O_2_** | 0.579 | 0.538 | 0.997 | 0.702 | 0.619 | 1.002 | 0.625 | 0.596 | 0.954 | 0.455 | 0.691 | 1.032 | 0.375 | 0.639 | 0.839 |
| **SOD** | 0.413 | 0.494 | 0.900 | 0.347 | 0.586 | 0.588 | 0.169 | 0.554 | 0.767 | 0.268 | 0.676 | 0.710 | 0.117 | 0.572 | 0.607 |
| **CAT** | 0.691 | 0.477 | 1.037 | 0.752 | 0.549 | 0.710 | 0.517 | 0.688 | 0.988 | 0.434 | 0.578 | 0.932 | 0.600 | 0.570 | 0.813 |
| **Total Chlorophyll** | 0.918 | 0.978 | 0.984 | 0.941 | 0.950 | 0.910 | 1.030 | 1.015 | 0.998 | 1.080 | 0.963 | 1.063 | 0.905 | 0.987 | 0.944 |
| **Chlorophyll a** | 0.923 | 0.978 | 0.991 | 0.950 | 0.951 | 0.920 | 1.031 | 1.018 | 1.020 | 1.083 | 0.971 | 1.085 | 0.918 | 0.992 | 0.966 |
| **Chlorophyll b** | 0.896 | 0.976 | 0.958 | 0.902 | 0.949 | 0.874 | 1.024 | 1.001 | 0.915 | 1.067 | 0.929 | 0.983 | 0.850 | 0.963 | 0.863 |
| **FRAP** | 1.067 | 0.996 | 1.084 | 1.015 | 0.939 | 1.164 | 0.874 | 1.044 | 0.986 | 0.925 | 0.908 | 0.934 | 0.808 | 0.939 | 0.878 |
| **Phosphomolybdate** | 1.048 | 1.011 | 1.032 | 1.037 | 1.002 | 1.014 | 0.959 | 1.016 | 0.969 | 0.949 | 0.958 | 0.940 | 0.931 | 0.997 | 0.933 |
